# Shaping Ultrafast Pulses for Enhanced Resonant Nonlinear Interactions

**DOI:** 10.1021/acs.nanolett.5c03349

**Published:** 2025-11-17

**Authors:** Omri Meron, Snir Nehemia, Uri Arieli, Haim Suchowski

**Affiliations:** † Condensed Matter Physics Department, School of Physics and Astronomy, Faculty of Exact Sciences, 26745 Tel Aviv University, Tel Aviv 6997801, Israel; ‡ Center for Light-Matter Interaction, 26745 Tel Aviv University, Tel Aviv 6997801, Israel

**Keywords:** Coherent Control, Pulse shaping, Ultrafast Physics, Nonlinear Optics, Plasmonic, LSPR

## Abstract

Coherent control with shaped ultrafast pulses is a powerful approach for steering nonlinear light–matter interactions. Prior quantum control studies show that, beyond transform-limited pulses, antisymmetric spectral phases can efficiently drive nonresonant multiphoton transitions. However, resonant multiphoton transitions experience dispersion from the material’s spectral-phase response, which lowers nonlinear efficiency. Preshaping the pulse to compensate for this response can restore and enhance nonlinear interactions beyond transform-limited pulses. But is this the only spectral phase to yield such enhancement? Here, we study sub-10 fs single-pulse four-wave mixing in resonant plasmonic nanostructures using arctangent spectral-phase-shaped pulses. We identify two distinct enhancement regimes: one compensates material dispersion, and a counterintuitive regime induces an antisymmetric polarization response that drives constructive multiphoton pathway interference. Our theoretical analysis elucidates both phenomena. Notably, it predicts that both mechanisms exhibit distinct scaling behaviors with harmonic order and quality factor, offering a new pathway for significantly enhancing resonant nonlinear processes.

## Graphical abstract

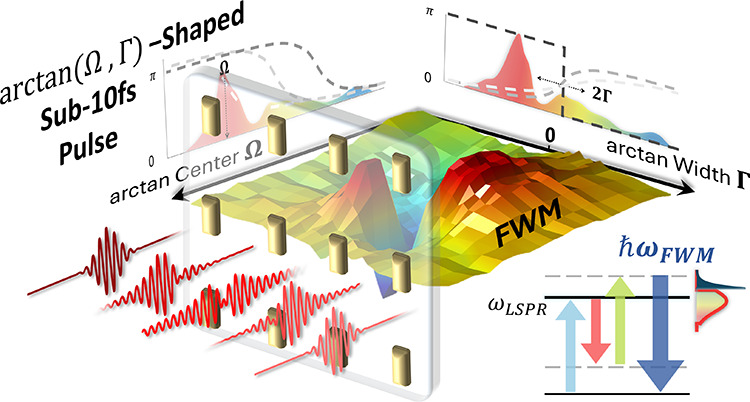

Coherent control using shaped ultrafast optical pulses has emerged as a powerful framework for steering quantum and nonlinear interactions in matter.^1^ By tailoring the spectral phase, amplitude, and polarization of femtosecond pulses,^2^ one can selectively manipulate excitation pathways with exceptional precision.^3–10^ Among these parameters, spectral phase plays a particularly central role, especially in resonant multiphoton processes, where interactions are inherently noninstantaneous and strongly influenced by the system’s spectral response.

A pivotal advance was made by the Silberberg group, who introduced the concept of quantum control of multiphoton transitions using shaped pulses in both nonresonant and resonant atomic media.^11^ In their seminal work, they showed that a pulse with a spectral phase antisymmetric around half the two-photon resonance frequency could efficiently drive multiphoton excitations, achieving results comparable to transform-limited (TL) pulses.^4,12^ This finding was strikingly counterintuitive: although such shaped pulses can exhibit extremely long temporal durations and very low peak power, they nonetheless produce maximal nonlinear responses due to the constructive interference of multiphoton pathways. Further, Silberberg and colleagues demonstrated that TL pulses are often not optimal for resonant multiphoton interactions, highlighting the need to tailor the spectral amplitude or phase to compensate for the system’s resonant response.^13^ In particular, they showed that applying a π-step spectral phase centered at the resonance frequency, shaping the incoming pulse to counteract the resonant spectral phase, enables temporal compression that leads to a transient enhancement of the induced transitions.^14^ These foundational insights established spectral phase shaping as a critical strategy for enhancing nonlinear interaction strengths and revealed the deep connection between phase symmetry, temporal dynamics, and quantum interference in multiphoton processes.

Originally demonstrated in isolated atomic and molecular systems, pulse shaping has since evolved into a versatile technique applied across diverse platforms, including nanostructures and condensed-matter systems.^15–20^ Stockman proposed extending these concepts to nanoplasmonics, showing that the temporal profile of excitation pulses could be tailored to match the time-reversed resonant dynamics of carriers in metallic nanoparticles.^21,22^ This strategy, aimed at localizing optical energy far below the diffraction limit, led to various theoretical and experimental demonstrations.^8,23–25^ Notably, Huang introduced a deterministic framework in which spectral phase functions derived from FDTD simulations were used to compress plasmonic responses in time, enabling control over ultrafast nanoscale fields.^26^ Recent experiments have reaffirmed the critical role of spectral phase in such regimes. For example, Bahar et al. demonstrated coherent control of second-order nonlinear enhanced emission in U-shape plasmonic resonance by optimizing chirped pulses, clearly revealing the noninstantaneous character of the interaction.^27^

While controlling resonant multiphoton interactions in complex systems remains challenging due to rapid decoherence and many-body effects, in our recent study on coherent control of two-dimensional semiconductors, we further advanced these ideas by demonstrating that in order to maximize the third-order nonlinear response near resonance, we introduced an arctangent (Atan) spectral phase function, precisely matched in center and width to the exciton resonance frequency and its decoherence rate. This function effectively counteracts the dispersion induced by the interaction of the 2D excitonic resonance, enabling a compressed temporal polarization response that enhances nonlinear optical performance. Importantly, this method allowed simultaneous compensation of multiple resonances within the pulse bandwidth, showcasing the strength of spectral phase-based coherent control in complex materials.^28^

Here, we systematically map the Atan spectral phase space of the ultrafast resonant plasmonic response by scanning its central frequency and spectral width. We employ shaped single-pulse, four-wave mixing (FWM) to probe χ^(3)^ processes. By mapping the nonlinear multiphoton enhancement and suppression landscape across the spectral phase space, we uncover a symmetric structure governed by detuning and phase parity. This phase-space topology reveals a well-established enhancement region where the spectral phase naturally compensates for the plasmonic resonant dephasing,^26–28^ allowing for direct extraction of the near-field resonant frequency and line width. More surprisingly, we identify a second, previously unexplored enhancement region that arises from antisymmetric phase relations, where the applied phase adds dispersion rather than compensates for the resonance-induced spectral phase, creating constructive multiphoton interference through a fundamentally different mechanism. This finding bridges insights from both resonant and nonresonant coherent control schemes, offering a unified framework for exploiting constructive multiphoton interference in complex media. To support these findings, we develop a compact second-order model that captures the observed enhancement patterns and show the approach extends to higher-order processes, providing significant enhancement factors for perturbative harmonic generation, depending on the pulse-resonance quality ratio (Q-ratio). Our results demonstrate that spectral phase symmetry and detuning fundamentally govern multiphoton efficiency in resonant systems, enabling rational design of shaped pulses for enhanced nonlinear processes.

In our experiments, we utilize a spatial light modulator (SLM)-based pulse shaping setup, as depicted in Figure 1. The SLM is positioned in the Fourier plane of a 4f system.^2^ This arrangement spatially disperses the sub-10 fs ultrabroadband pulse into aligned spectral components. The SLM enables precise temporal shaping of the spectral phase, ϕ_*SLM*_(λ), as illustrated in Figure 1.b. Additionally, the Fourier plane serves as a sharp edge filter which helps truncate the blue end of the spectrum. Upon passing through a tightly focusing mirror objective (Pike, NA-0.78), the shaped and truncated pulse interacts with an array of gold nanobars. The pulse is linearly polarized along the nanobars’ elongated axis. The reflection spectrum of the nanobars, as measured with our ultrabroadband pulse (gray shaded area in Figure 1c), displays a pronounced resonance peak at ω_*LSPR*_ = 1.68 eV = 738 nm, verifying the effective photoexcitation of the localized surface plasmon resonance (LSPR) mode. Importantly, the spectral bandwidth of the pulse is substantially broader than the LSPR line width, facilitating the simultaneous excitation across the entire bandwidth of the metallic nanostructure’s LSPR response. This is crucial for achieving coherent control over the excitation.

**Figure 1:**
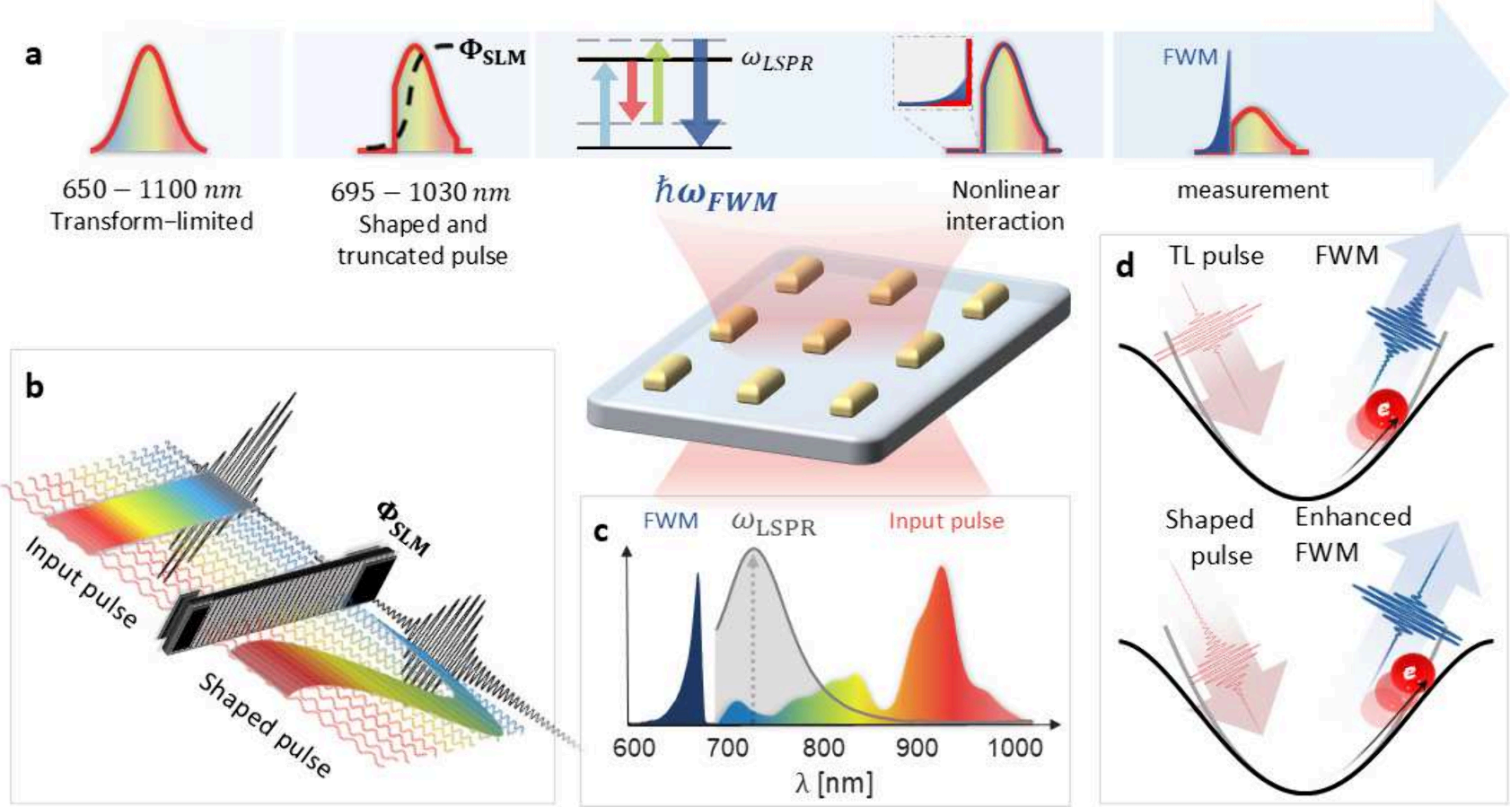
(a) A diagram of the single-pulse FWM experimental apparatus in the frequency domain: a sub-10 fs pulse is shaped and spectrally truncated by the SLM (see (b)). The pulse is then focused onto an array of gold nanobars, generating a FWM nonlinear response that is detected by a spectrometer via reflection. (b) Schematic illustration of a negative Atan spectral phase applied by the SLM, and how it modifies the wavelength-dependent group-delay. (c) Power spectral density (colorful area) of the ultrabroadband sub-10 fs input pulse used in the experiment, overlaid with the linear reflection spectrum of the gold nanobar array (gray), acquired using the same excitation pulse. The resulting FWM signal (dark blue), collected in reflection and scaled for visibility, appears on the anti-Stokes side of the spectrum. (d) Illustration of the quartic correction to the harmonic potential, giving rise to third-order nonlinearity from the LSPR. A shaped driving pulse can increase the transient oscillator displacement, optimizing nonlinearity compared to a TL pulse.

Beyond linear interactions, the ultrabroadband pulse also drives various intrapulse nonlinear wave-mixing processes. Due to interband absorption and the centrosymmetric geometry of the gold nanobar, sum-frequency generation (SFG) was not observed. Instead, we focus our measurements on the FWM signal, which arises from the coherent nonlinear interaction of three frequency components within the pulse, combining along distinct optical pathways with well-defined phase relationships. This FWM signal is collected in reflection on the anti-Stokes side of the pulse, outside the truncated pulse spectrum, with the fundamental attenuated using a short-pass edge filter. The experimental apparatus and FWM detection scheme are depicted in Figure 1.^28–31^ We use the SLM to systematically scan the full parameter space of the Atan phase function, scanning both the central frequency Ω and line width Γ:

1 $$\phi_{E}\left(\omega \right)=\tan^{-1}\left(\frac{2\Gamma \omega }{\Omega^{2}-\omega^{2}}\right)$$

This parametrization allows us to tailor the spectral phase to either compensate or add dispersion to the intrinsic resonant phase of the system. As shown in Figure 2a, the obtained two-dimensional FWM intensity map reveals a rich landscape of multiphoton pathway interference responses. First, we observe the primary enhancement FWM region in Figure 2a, which closely corresponds in central frequency to the LSPR Ω = ω_*LSPR*_ = 1.68 eV, whereas the optimal line width, Γ = – γ_*LSPR*_ = 0.049 ± 0.015 eV, is notably narrower (in absolute value) than the line width measured in linear reflection, $\gamma_{LSPR}^{lin}=0.99\;eV$ , using the same ultrabroadband laser source (see Supporting Information). These results highlight two key differences from far-field linear measurements: (i) the measured FWM signal reflects localized near-field resonant properties that can deviate from spatially averaged far-field linear responses,^32–34^ and (ii) the sub-10 fs nonlinear response probes the system near its homogeneous broadening limit, effectively filtering out slower inhomogeneous effects.

**Figure 2:**
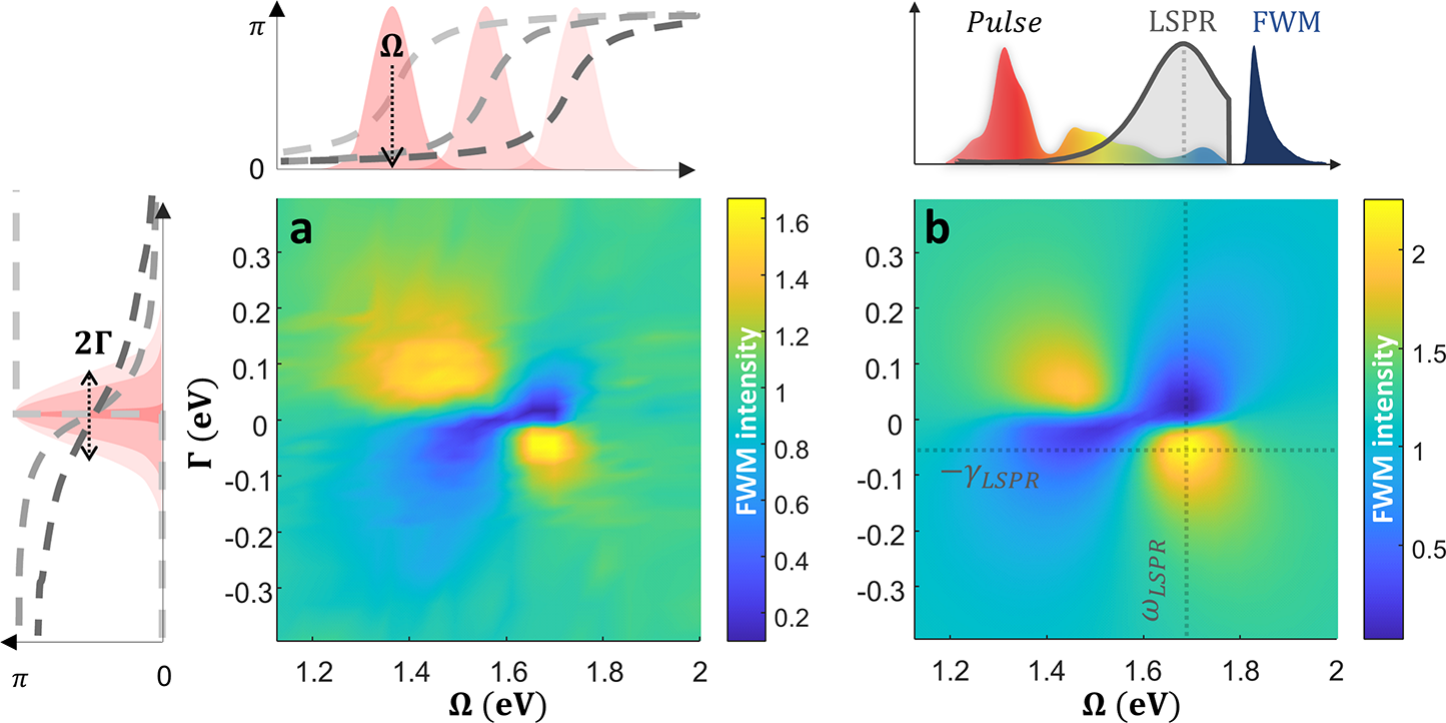
Measured and simulated 2D landscapes of the Atan phase-space scan. (a) A measurement of the integrated FWM intensity (normalized to the TL case) as a function of the Atan spectral phase parameters: line width Γ and central frequency Ω, as defined in eq 1. Illustration of an Atan phase, applied by the SLM, while varying Γ for a given Ω (left panel) and vice versa (top panel). At the edges of the measurement, the phase approaches the TL case. (b) Simulated 2D phase-space map of the integrated FWM response based on the AHO model (eq 4). The top panel includes the spectral bandwidth of the input pulse, the position of the LSPR, and the measured FWM signal, all plotted on the same energy scale as the Ω axis.

The measured phase landscape in Figure 2a also reveals intriguing secondary regions of enhanced and suppressed nonlinear responses. To disentangle the multiphoton interference effects underlying our experimental observations, we model the nonlinear dipolar LSPR response using an anharmonic oscillator (AHO) framework, extending the classical harmonic description of electron displacement by incorporating quadratic, cubic,^33^ and higher-order nonlinearities.^35^ The electron displacement *x*(*t*), driven by the ultrafast electric field *E*(*t*), is characterized by the following equation:

2 $$ẍ\left(t\right)+2\gamma_{0}ẋ\left(t\right)+\omega_{0}^{2}x\left(t\right)+\overset{\infty }{\underset{n=2}{\sum }}\alpha_{n}x^{n}\left(t\right)=-\frac{eE\left(t\right)}{m}$$

where ω_0_ and γ_0_ are the LSPR resonance frequency and line width, respectively, and α_*n*_ are the n^th^ order nonlinear coefficients. Concentrating on third-order nonlinearity (*n* = 3) and assuming a perturbative regime, we decompose the displacement as *x*(*t*) = *x*_0_(*t*) + *δx*(*t*), where *x*_0_(*t*) is the linear (Lorentz) oscillator solution, and *δx*(*t*) is the nonlinear correction. In the frequency domain, the linear response is

3 $${\mathrm{x̃}}_{0}\left(\omega \right)\propto Ẽ\left(\omega \right)D\left(\omega \right)e^{i\left(\phi_{E}+\phi_{D}\right)}$$

with $D\left(\omega \right)={\left(\omega_{0}^{2}-\omega^{2}-2i\omega \gamma_{0}\right)}^{-1}$ . Thus, the third-order nonlinear correction will scale as a 3-fold autoconvolution:

$$\delta {\mathrm{x̃}}^{\left(3\right)}\left(\omega \right)\approx -\alpha_{3}D\left(\omega \right)\left[{\mathrm{x̃}}_{0}(\omega )*{\mathrm{x̃}}_{0}(\omega )*{\mathrm{x̃}}_{0}\left(\omega \right)\right]$$ 4

Differing from narrow-band source treatments, which simplify convolution to a sum over selected discrete mixing frequencies,^35^ we focus on retaining all intrapulse four-wave interactions within our ultrabroadband source.

From eq 3, we can observe that the nonlinear polarization $P^{\left(3\right)}\propto |\delta {\mathrm{x̃}}^{\left(3\right)}\left(\omega \right){|}^{2}$ is maximized when ${\mathrm{x̃}}_{0}\left(\omega \right)=\left|{\mathrm{x̃}}_{0}\left(\omega \right)\right|$ . To achieve this, the electric field phase can be tailored to counteract the resonant phase in eq 3 by setting $\phi_{E}\left(\omega \right)=-\phi_{D}\left(\omega \right)=-\tan^{-1}\left(\frac{2\gamma_{0}\omega }{\omega_{0}^{2}-\omega^{2}}\right)$ , leading to an optimally compressed (i.e., TL) oscillator displacement.^26,28^ Moreover, the Atan phase enhances the instantaneous displacement of the oscillator. As the oscillator is driven away from its minimum energy, the potential becomes less quadratic, thereby increasing the nonlinear polarization (see illustration in Figure 1.d).

Figure 2b shows the simulated FWM response from our AHO model as a function of the Atan phase parameters Ω and Γ, using the same driving pulse and LSPR parameters extracted from the experiment. Remarkably, the simulated FWM response reproduces the experimental landscape with striking accuracy, capturing the primary enhancement peak centered at resonance and reflecting the structure of the secondary enhancement and suppression regions. Both experimental and simulated maps display a characteristic four-quadrant pattern, where each quadrant corresponds to a distinct regime of multiphoton pathway interference. However, while the AHO model quantitatively matches the data, the physical intuition behind the secondary enhancement remains elusive. This motivates our turn to a simpler nonlinear model, which offers clearer insight into the symmetry and interference features observed in the phase-space landscape.

We simulate the Atan phase-space response of a second-order nonlinear oscillator (setting *n* = 2 in eq 2), focusing on SFG under a 6 fs Gaussian driving pulse with carrier frequency ω_*c*_. Figure 3a displays the resulting nonlinear response at the fixed detection second harmonic frequency 2ω_*c*_. Similar to the line width Γ axis, which is scanned from negative to positive values, effectively reversing the sign of the imposed Atan spectral phase, we set the frequency Ω axis relative to ω_*c*_. This representation naturally defines a coordinate system centered on two symmetry axes: (i) a vertical line at Ω = ω_*c*_, which roughly distinguishes between on/off resonance excitation, and (ii) a horizontal line at Γ = 0, which separates phase functions that compensate (negative Γ) or add dispersion (positive Γ) to the intrinsic phase of the resonance.

**Figure 3:**
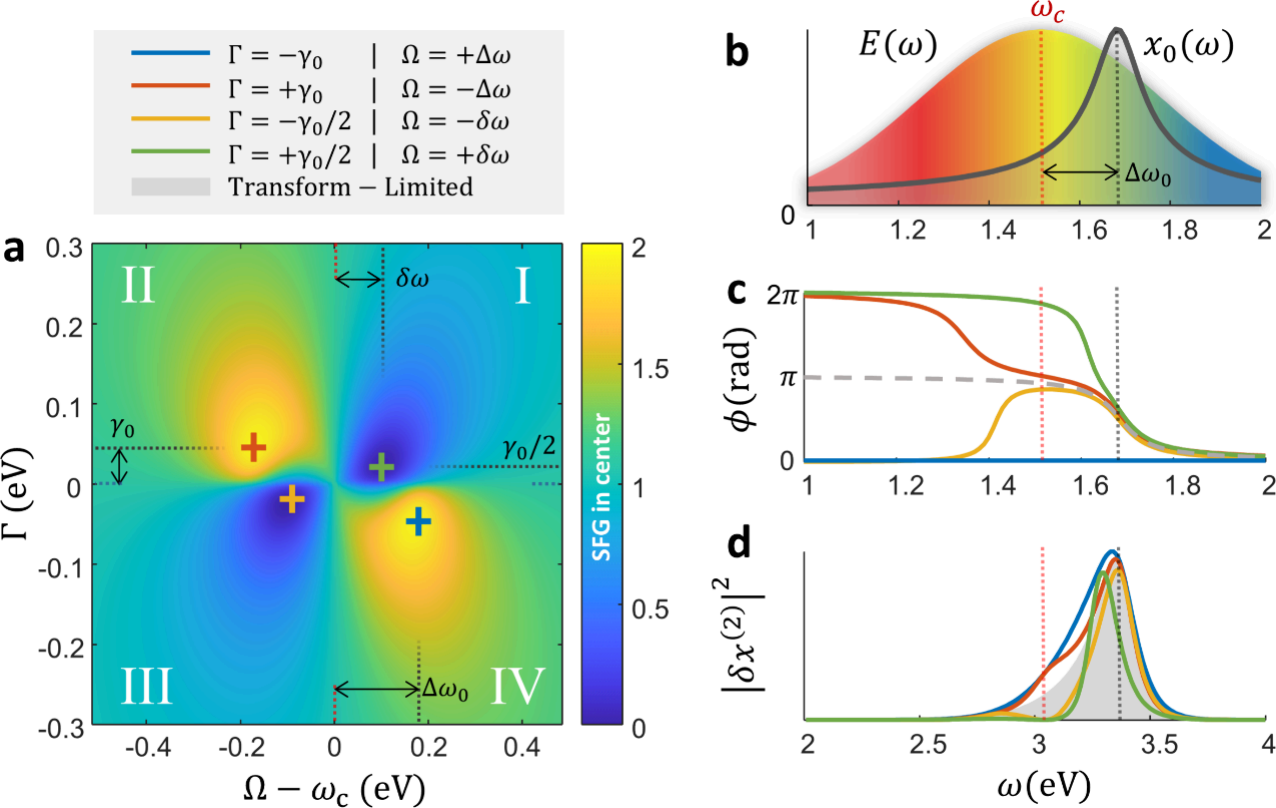
Theoretical analysis of multiphoton pathway interference using a second-order nonlinear model. (a) Simulated SFG intensity at fixed detection frequency 2ω_*c*_ (normalized to the TL case) as a function of Atan phase center (Ω – ω_*c*_) and line width Γ, revealing a characteristic four-quadrant symmetry. (b) Spectral profiles of the driving Gaussian field *E*(ω) and a blue-detuned Lorentzian resonance *x*_0_(ω). (c) Spectral phase of the oscillator displacement *x*_0_(ω) for selected points in panel (a). (d) Simulated SFG power spectral density |*δx* ^(2)^(ω)|^2^ corresponding to the configurations in panel (c), highlighting broadening and narrowing of the spectrum via constructive and destructive multiphoton pathway interference.

Two quadrants in the phase-space, II and IV, exhibit clear enhancement in the SFG signal relative to the TL case. In quadrant IV, the enhancement peaks when the applied Atan phase compensates the resonant phase ϕ_*E*_(Ω, Γ) = – ϕ_*D*_(ω_*c*_ + Δω_0_, γ_0_), yielding a temporally compressed oscillator displacement $\left(\phi_{x_{0}}\left(\omega_{c}+\Delta \omega_{0},-\gamma_{0}\right)=const\right)$ and maximal nonlinear response. In contrast, the enhancement in quadrant II originates from a different mechanism. Here, the applied spectral phase adds dispersion to the intrinsic resonance phase, resulting in an overall antisymmetric displacement phase profile around ω_*c*_, i.e., $\phi_{x_{0}}\left(\omega_{c}-\Delta \omega_{0},\gamma_{0}\right)=-\phi_{x_{0}}\left(\omega_{c}+\Delta \omega_{0},\gamma_{0}\right)$ (ignoring global phases). Similar antisymmetric phases are known to preserve two-photon absorption efficiency in nonresonant excitations,^4^ yet their effect in resonant scenarios has not been previously reported. The antisymmetric displacement phase profile ensures that photon pairs symmetric about the carrier frequency ω_*c*_ experience identical group delays when interacting with the resonance, i.e., τ_*g*_(ω_*c*_ + Δω) = τ_*g*_(ω_*c*_ – Δω). This group delay symmetry preserves the phase relationships necessary for constructive interference, as frequency-symmetric photon combinations arrive simultaneously despite the dispersive resonant medium. Consequently, this antisymmetry leads to constructive interference among all two-photon pathways leading to 2ω_*c*_, and manifests in spectral broadening of the SFG response (Figure 3d). In the remaining quadrants (I and III), the spectral phase distorts this symmetry, resulting in destructive interference and suppressed nonlinear output, consistent with previously described ’dark pulse’ conditions.^11^ This phase-space symmetry suggests a general design principle: tailoring the spectral phase to enforce antisymmetry (or group-delay symmetry) enables steering the system toward regimes of constructive multiphoton interference. While this understanding emerges from a second-order model, it offers predictive value for more complex higher-order processes, as shown in the FWM response of Figure 2.

Having demonstrated how tailored Atan spectral phases modulate FWM and SFG through coherent multiphoton interference, we extend our analysis to higher-order nonlinear processes where these enhancement mechanisms become increasingly pronounced. We investigate harmonic generation up to seventh order, under resonant excitation, where spectral phase effects remain largely unexplored despite their potential for addressing the exponentially diminished efficiencies characteristic of higher-order harmonics.^36–40^ Our classical perturbative simulations based on eq 2 capture the essential multiphoton pathway interference physics while remaining within the weak-field regime. The importance of resonantly enhanced harmonic generation has been established across diverse platforms operating in the perturbative regime, from plasmonic nanostructures to all-dielectric metasurfaces.^27,39,41,42^

Figure 4a reveals the pronounced effects of our two distinct phase strategies on high harmonic generation. For a lossy resonance, characteristic of plasmonic systems, both the phase-compensating Atan function (AT, blue) and the antisymmetric phase configuration (AS, orange) yield dramatic enhancement and spectral broadening of harmonic signals compared to the transform-limited reference (gray). Notably, both phase shaping strategies show an exponential increase in enhancement with harmonic order. Interestingly, the antisymmetric approach achieves substantial enhancement even though it introduces dispersion, rather than canceling it as in the phase-compensating strategy. The spectral broadening observed in both cases reflects the constructive interference among multiple multiphoton pathways, with each strategy enforcing different symmetry conditions that preserve phase relationships across the broad harmonic spectrum.

**Figure 4:**
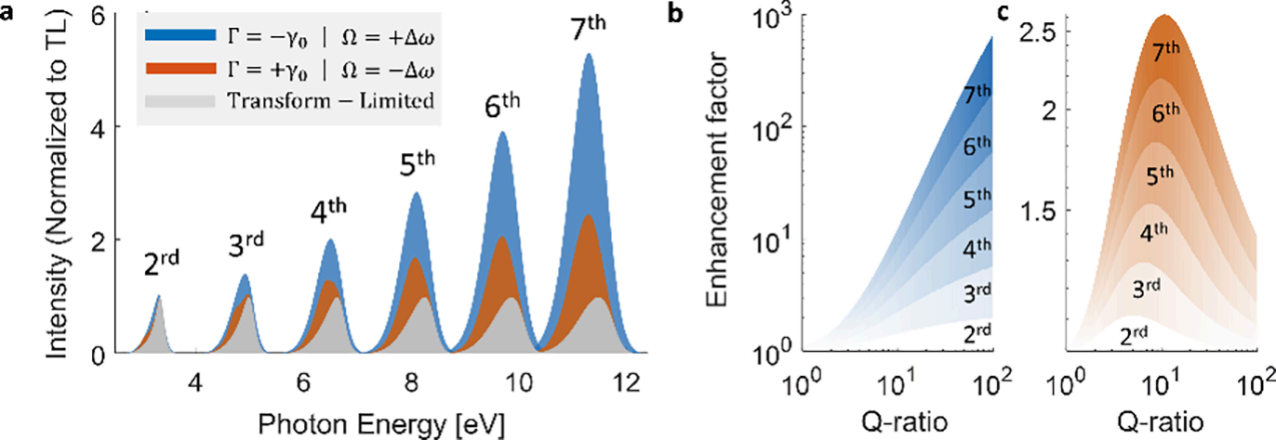
Simulated harmonic generation enhancement (relative to the TL case) through tailored Atan spectral phases and Q-ratio scaling. (a) Harmonic generation spectra comparing phase-compensating Atan function (blue, Γ = −γ_0_|Ω = +Δω) and antisymmetric phase configuration (orange, Γ = +γ_0_|Ω = −Δω) with transform-limited reference (gray). Both approaches yield enhancement and spectral broadening of harmonic signals up to the seventh order. (b) Enhancement factor scaling with Q-ratio for the phase-compensating approach, showing exponential improvement with increasing Q-ratio across all harmonic orders. (c) Enhancement factor scaling with Q-ratio for the antisymmetric approach, reaching optimal performance at intermediate Q-ratios. Both strategies demonstrate that enhancement factors increase dramatically with harmonic order, with the scaling behavior strongly dependent on the pulse-resonance quality ratio.

To establish the universal applicability of these findings beyond specific material parameters, we introduce the Q-ratio, the ratio between resonance line width γ_0_ and driving pulse bandwidth, as a fundamental design parameter. This dimensionless quantity encapsulates the essential pulse-resonance interplay and enables direct translation of our results to any combination of driving pulse and resonant system. As demonstrated in Figures 4b,c, the scaling behaviors of our two enhancement mechanisms exhibit distinctly different dependencies on this universal parameter. The phase-compensating approach shows incredible improvement with increasing Q-ratio, consistent with the efficient dispersion cancellation in higher-quality resonances. In contrast, the antisymmetric strategy reaches optimal performance at intermediate Q-ratios (5̃-10) before it reduces, reflecting the balance required between maintaining antisymmetric phase relationships and avoiding excessive dispersion. This Q-ratio framework provides a powerful predictive tool for optimizing nonlinear coherent control across diverse physical platforms under resonant excitation.

In conclusion, we have demonstrated a deterministic coherent control strategy for enhancing nonlinear optical processes in resonant plasmonic nanostructures using tailored Atan spectral phases. This approach uncovers a symmetric phase-space landscape of enhancement and suppression, governed by the interplay between spectral detuning and phase parity. We identify two distinct enhancement mechanisms: phase compensation that shows strong enhancement with increasing Q-ratio, and antisymmetric shaping that optimizes at intermediate Q-ratios, both exhibiting exponential enhancement trends with increasing harmonic order. The compensating phase is expected to compress all harmonic orders by counteracting the resonant dispersion, leading to constructive temporal overlap across the entire harmonic spectrum (assuming the nonlinear frequency generation is far from resonance). Crucially, the underlying methodology is general and can be extended not only to arbitrarily shaped nanoparticles but to any resonant system that follows the classical anharmonic dynamics derived in our model.^28^ The introduction of the Q-ratio as a universal design parameter enables predictive mapping of pulse-resonance interplay across diverse platforms, with enhancement factors that rise with harmonic order, resonance quality factor, and driving pulse bandwidth.

Notably, our sub-10 fs single-pulse apparatus and phase-selective nonlinear measurements enable access to the near-field LSPR response approaching its homogeneous limit, effectively filtering out inhomogeneous broadening contributions that typically obscure resonant parameters in linear far-field spectroscopy.^43,44^ Furthermore, the emergence of the nonintuitive antisymmetric enhancement mechanism improves nonlinear generation efficiency while sustaining low peak power for dispersed pulses, offering a complementary pathway to conventional phase compensation strategies.

Looking forward, it would be particularly interesting to apply this 2D phase-space approach to more complex resonant systems, such as coupled nanoresonators,^45–47^ Fano resonances,^48,49^ BICs (Bound states in the Continuum),^50^ or dark-bright mode hybrids.^51,52^ Using this method, we may be able to disentangle the rich interference phenomena in such systems and thereby unravel the underlying microscopic mechanisms driving their nonlinear responses.^28,53^ The coherent spectral phase shaping approach demonstrated here not only optimizes nonlinear conversion efficiency but also holds significant promise for enhancing spatial resolution and selectivity in nonlinear near-field imaging, by enabling dynamic control over nanoscale energy distribution.^7,21,22^

## Supplementary Material



## Notes

SEM images and linear reflection spectrum fitting in supplementary notes S1 and S2 and Figures S1 and S2 (PDF)
