# Supplementary material for: Shaping Ultrafast Pulses for Enhanced Resonant Nonlinear Interactions
Source: Nano Lett. 2025 Nov 17;25(47):16614–20. doi: 10.1021/acs.nanolett.5c03349 (PMC12670494; doi:10.1021/acs.nanolett.5c03349)
Supplement: Supplementary file 1 [file nl5c03349_si_001.pdf]

Supplementary Information –

Shaping Ultrafast Pulses for Enhanced Resonant Nonlinear Interactions

Omri Meron[1,2], Snir Nehemia[1,2], Uri Arieli[1,2], and Haim Suchowski[1,2†]

[1]Condensed Matter Physics Department, School of Physics and Astronomy, Faculty of Exact Sciences, Tel Aviv University, Tel-Aviv, 6997801, Israel.
[2]Center for Light-Matter Interaction, Tel Aviv University, Tel-Aviv, 6997801, Israel

Table of Contents

S1 SEM characterization..2
S2 Linear characterization..2

S1 SEM characterization

The sample consists of a square lattice of widely spread Au nanoparticles with a spacing of 450nm, placed on top of an SiO2 substrate. Scanning electron microscope (SEM) images are presented In figure S1. The measured particle size is about 110nm long, 35nm wide and 40nm high.

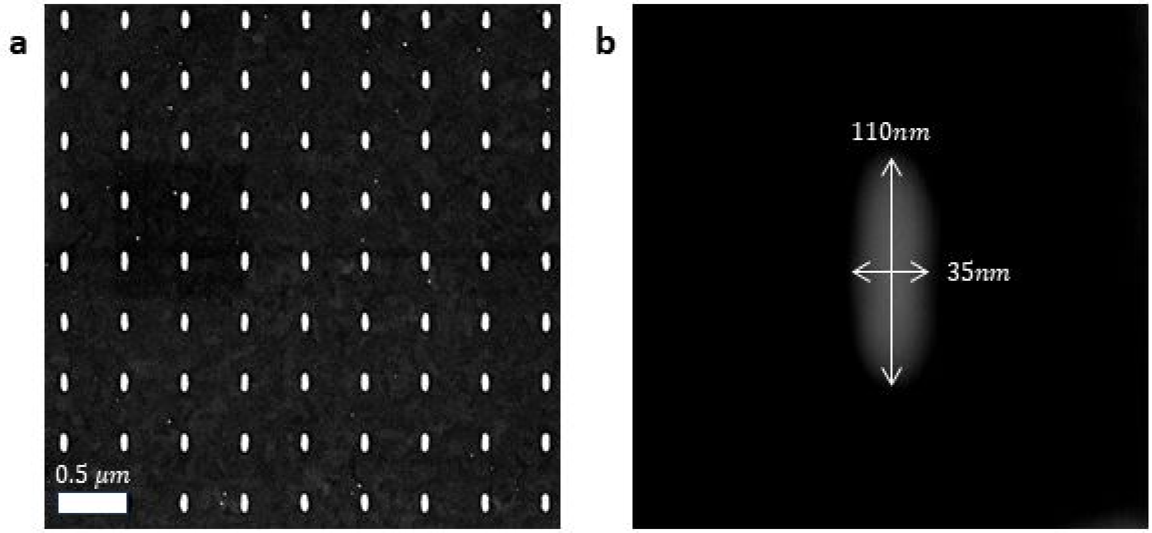

Figure S1 *SEM images of Au nanobars on a SiO2 substrate. (a) a square lattice of single nanobars with 450 nm spacing. (b) A single nanober close-up.*

S2 Linear characterization

To characterize the specific array of interest we conducted a reflection measurement using our ultrabroadband laser pulse (650-1030nm). To extract the resonant parameters of the localized surface plasmon resonance (LSPR) we fitted the measured spectrum to the scattering cross-section σ_{scatt} of a damped harmonic oscillator with displacement x_0:

$$\sigma_{scatt} \propto \omega^4 |x_0|^2$$

The accumulated spectrum and fit are displayed in figure S2.

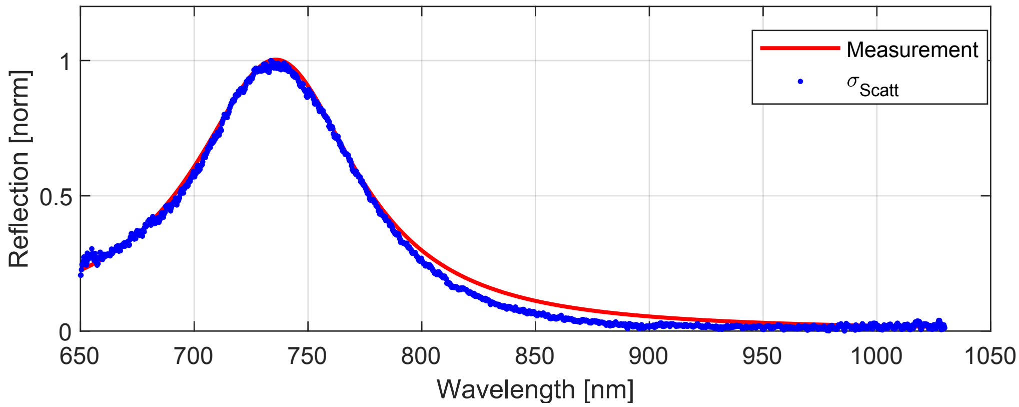

***Figure S2** Normlized reflection spectrum (blue) and fit (red) of the nanobars. The measurement was obtained using the sub-10 fs ultrabroadband laser pulse.*

The extracted LSPR frequency and damping parameters are $\omega_{LSPR} = 1.679\ [eV] = \ 738\ [nm]$, γ $= 98.6 \pm 1.3\ [meV]$.
